# Development and Validation of an HPLC-ELSD Method for the Quantification of 1-Triacontanol in Solid and Liquid Samples

**DOI:** 10.3390/molecules23112775

**Published:** 2018-10-26

**Authors:** Stefania Sut, Clizia Franceschi, Gregorio Peron, Gabriele Poloniato, Stefano Dall’Acqua

**Affiliations:** 1 Department of Agronomy, Food, Natural Resources, Animals and Environment (DAFNAE), Agripolis Campus, University of Padova, 35020 Legnaro, Italy; stefania_sut@hotmail.it; 2 ILSA S.P.A., Via Quinta Strada, 28, 36071 Arzignano, Italy; cfranceschi@ilsagroup.com; 3 Department of Pharmaceutical and Pharmacological Sciences, University of Padova, Via Francesco Marzolo, 5, 35131 Padova, Italy; gregorio.peron@studenti.unipd.it (G.P.); gabriele.poloniato@unipd.it (G.P.)

**Keywords:** 1-triacontanol, HPLC-ELSD, biostimulant, method validation

## Abstract

1-Triacontanol (TRIA) is gaining a lot of interest in agricultural practice due to its use as bio-stimulant and different types of TRIA-containing products have been presented on the market. Up to date, TRIA determination is performed by GC analysis after chemical derivatization, but in aqueous samples containing low amounts of TRIA determination can be problematic and the derivatization step can be troublesome. Hence, there is the need for an analysis method without derivatization. TRIA-based products are in general plant extracts that can be obtained with different extraction procedures. These products can contain different ranges of concentration of TRIA from units to thousands of mg/kg. Thus, there is the need for a method that can be applied to different sample matrices like plant materials and different plant extracts. In this paper we present a HPLC-ELSD method for the analysis of TRIA without derivatization. The method has been fully validated and it has been tested analyzing the content of TRIA in different dried vegetal matrices, plant extracts, and products. The method is characterized by high sensitivity (LOD = 0.2 mg/L, LOQ = 0.6 mg/L) and good precision (intra-day: <11.2%, inter-day: 10.2%) being suitable for routine analysis of this fatty alcohol both for quality control or research purposes.

## 1. Introduction

1-Triacontanol (TRIA), a fatty alcohol composed of 30 atoms of carbon, acts as a natural growth regulator in plants. It can be found in the epicuticular waxes of a widely diverse range of genera, such as California croton (*Croton californicus*), blueberry (*Vaccinium ashei*), Brazilian palm (*Copernicia cerifera*), runner bean (*Phaseolus multiflorus*), white clover (*Trifolium repens*), alfalfa (*Medicago sativa*) and in physic nut (*Jatropha curcas*), for example. It is used to enhance the crop production in millions of hectares, particularly in Asia [1,2,3,4,5,6,7,8]. Several researchers have reported the TRIA-mediated improvement of several parameters in various crops, such as growth, yield, photosynthesis, protein synthesis, uptake of water and nutrients, nitrogen-fixation, enzymatic activities and contents of free amino acids, reducing sugars, soluble proteins, and active constituents as essential oil. Furthermore, TRIA could enhance the physiological efficiency of the cells and, thus, could exploit the genetic potential of the plants to a large extent [1,9,10].

To assess the effects of TRIA-containing products on plants, accurate determinations of the concentration of TRIA and quantification of the doses are needed. Several published methods for the analysis of fatty alcohols are based on GC approaches that include a derivatization step, which is required to enhance the volatility of these compounds. Recently, a method for the determination of TRIA and other lipophilic constituents in vegetables based on saponification, liquid-liquid extraction and derivatization with TMS and GC-MS analysis was proposed. Despite the sensitivity and specificity that can be achieved with GC-MS, the main drawbacks of these determinations are related to the derivatization procedure and to the relative high cost and complex management of GC-MS equipment [5,11,12,13,14]. Furthermore, although sample derivatization can be quite easily managed for dried vegetable samples, it can be difficult for aqueous solutions or for complex formulated products containing emulsifiers, since additional drying steps are required. Furthermore, the methods using derivatization can suffer from the presence of a strong matrix effect, due to the fact that also interfering constituents can react with the silanization or esterification reagents. Liquid chromatography (LC)-based techniques may offer the opportunity to perform chromatographic analyses without derivatization.

Few data about the HPLC analysis of TRIA have been found in literature to date [6]. This may be addressed to the fact that TRIA does not contain a chromophore in its structure, hence UV detection could not be used. An alternative to overcome this issue and to avoid MS spectrometry is the use Refractive Index (RI) or Evaporative Light Scattering Detection (ELSD). ELSD offers the opportunity to use gradient elution and, to the best of our knowledge, no specific application to the analysis of TRIA has been reported in literature yet. In fact, only Hwang and Coll [6]. proposed an HPLC-ELSD method for the analysis of total policosanols in grain sorghum kernels and dried distilled grains, using silica as stationary phase. However, the authors reported that the separation of policosanols was not sufficient, hence their characterization was performed by GC. Due to the diffusion of TRIA-containing products for agricultural purposes, the development of a validated analytical method that allows its determination in different types of matrices is increasingly needed. As a matter of fact, TRIA may be present in these products at concentrations of 10–1000 ppm or dispersed in water for fertilization at low concentrations (10–50 ppm). Furthermore, it may be obtained in high amounts (up to 1–3% of dried extracts) by lipophilic extraction from vegetable matrices. For quantitative purposes and for the development of the method we used as plant source of TRIA *Medicago sativa*. Dried plant materials, enzymatic extracts, supercritical CO_2_ as well as pure TRIA were used as samples.

In this paper we present a new approach for the analysis of TRIA in plant materials and in formulated products using 5-α-cholestane (5AC) as internal standard (IS). The method, which allows the determination of TRIA up to 0.6 mg/L, doesn’t involve any derivatization neither the use of mass spectrometry. Extraction, ELSD parameters and chromatographic conditions were optimized and validated. The method is easily applicable and compared to conventional GC approaches allow direct analysis of TRIA without derivatization.

## 2. Results and Discussion

The method allows the determination and quantification of TRIA in several types of matrices, being useful for the analysis of the different types of products used in the agricultural field. The method is sufficiently sensitive to allow to detect TRIA in aqueous solutions up to 0.6 mg/L and is feasible also for the analysis of pure materials or highly concentrated lipophilic extracts. GC-based methods require derivatization [5,6,11,12], which can be problematic in the presence of heavy matrices containing interfering compounds that can react with the derivatizing agents. Furthermore, liquid products containing low amounts of TRIA (as many biostimulant formulations that are nowadays present on the market) may be critical for GC sample preparation due to high water contents or due to the presence of other formulants like surfactants. Thus, the proposed approach can be useful for the analysis of such products. Due to sensitivity, simple dilutions can be performed, if the sample contains sufficient TRIA amounts. On the other hand, extraction with dichloromethane in the presence of ISTD can be used with any type of liquid product and formulations. In the proposed method we used the Evaporative Light Scattering Detector (ELSD), which is a cheap instrument widely available on the market and easier to use compared to mass spectrometry. Furthermore, compared to refractive index detectors, ELSD is more versatile, given the possibility to perform gradient elutions that allow one to improve sample separation. Compared with a previously published HPLC method [6] the approach described in the present work uses a reverse phase column instead of direct phase chromatography.

### 2.1. Extraction of TRIA from Dried and Liquid Materials

Due to the lipophilic nature of TRIA, dichloromethane was revealed to be the most suitable solvent for its extraction from different matrices. Extraction from liquid samples can be performed by liquid/liquid partition, while extraction from plant material requires a 15 min extraction in an ultrasoonic bath. Due to the poor solubility of TRIA in water, its concentration in solution can be low. However, using surfactants, suspensions or colloidal suspensions can be obtained, and higher concentrations of TRIA in water could be achieved.

### 2.2. Specificity, Linearity, LOQ and LOD

**Figure 1:**
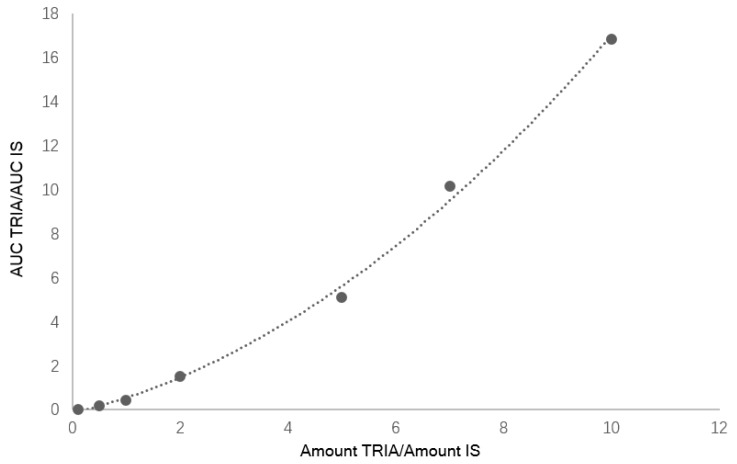
Calibration curve for TRIA.

Seven calibration mixtures prepared mixing different ratios of TRIA/IS (see Table 1) were used to create a calibration curve with a quadratic behavior in the considered calibration range (Figure 1). The obtained curve was *y* = 0.441*x*^2^ + 0.8212*x* + 0.004. The retention times of standards of TRIA (8.9 min) and 5AC used as IS (11.5 min) allowed the identification of compounds. LOD and LOQ for TRIA were 0.2 mg/L and 0.6 mg/L, respectively.

### 2.3. Recovery, Accuracy and Precision

To estimate the recovery of TRIA two different sets of samples were prepared. *Echinacea* root was used as dried vegetal material for recovery test due to the non-detectable content of TRIA. Table 2 reports the results related to recovery. The mean recovery of TRIA in Echinacea was 98.7%. The TRIA amounts used for spiking were ranging from 120 to 540 µg/g. Different Medicago sativa samples were extracted and assayed with and without spiking. The results, reported in Table 3, showed recovery ranging from 98.3% (spiked samples) to 100% (non-spiked samples). Furthermore, solutions containing different TRIA concentrations ranging from 5 to 100 mg/L of TRIA were prepared and analyzed (Table 3).

Precision was evaluated by analyzing TRIA samples spiked at four concentration levels, five times within the same day (intra-day precision) as well as on two consecutive days (inter-day precision). Results are reported in Table 2. Relative standard deviations (RSDs) varied in the range 1.7–11.2% and 1.1–10.2% for the intra-day and inter-day precision, respectively, being within the acceptance criteria of FDA [15].

### 2.4. Method Application

The robustness of the extraction protocol and of the analytical method were tested analyzing the different TRIA contents of dried samples of *M. sativa* leaves and leaves and stems. Furthermore, extracts obtained by supercritical CO_2_ containing 5000 and 28,000 mg/kg of TRIA were analyzed. Enzymatic extracts containing 10 mg/kg of TRIA were quantified, as well as enzymatic extracts with added TRIA at final concentrations of 15 and 40 mg/kg. A comparison of the amounts revealed by GC-MS [12] and the developed HPLC-ELSD method is reported in Table 4. The measured values were comparable between the different methods, showing that HPLC-ELSD is a suitable technique for the analysis of TRIA in different matrices.

## 3. Conclusions

The proposed method allowed the analysis of TRIA in liquid and solid samples and in different amounts without the need of derivatization, contrary to what is required for the GC analysis method. This can be an advantage compared to traditional GC methods, especially for the analysis of non-anhydrous samples like water-based liquids, that are among the most diffused biostimulants used in agricultural practice. The HPLC-ELSD approach appears to be precise, specific and sufficiently sensitive for the need of TRIA analysis in agricultural applications.

## 4. Experimental

### 4.1. Solvents and Materials

1-Triacontanol (TRIA), 5α-cholestane (5AC) and the silanization reagent (Sil-A) were purchased from Sigma Chemicals Co. (Milan, Italy). Sodium hydroxide (NaOH), hydrochloric acid (HCl), ethanol and dichloromethane were obtained from Merck KGaA (Darmstadt, Germany). HPLC-grade methyl *tert*-butyl ether, acetonitrile and methanol were obtained from Scharlab (Barcelona, Spain). Plant materials, supercritical CO_2_ extracts and enzymatic extracts from *Medicago sativa* L. were kindly gifted by the ILSA group S.P.A. (Vicenza, Italy).

### 4.2. Preparation of Standard Solutions

The development and validation of the procedure were carried out in model samples subjected to the procedure described below. 5AC was used as internal standard (IS), whereas 1-triacontanol was the target analyte. Stock solutions were prepared by dissolving 3 mg of the analytes in 10 mL of dichloromethane. Samples for calibration curves were finally prepared by diluting aliquots of stock solution to yield concentrations in the range of 10–100 µg/mL.

### 4.3. Preparation of Samples

The samples were weighted on the basis of the expected triacontanol content. Detailed procedures depending on the different types of starting materials are reported below.

#### 4.3.1. Dried or Fresh Plant Material, Solid Products

For plant material or formulated solid products containing less than 0.1% of TRIA, 1000 mg of material were weighted, added of the IS solution (1000 µL of a 500 µg/mL solution or absolute amounts of 300 to 500 μg of IS) and extracted in a flask with 50 mL of dichloromethane. Extraction was performed in ultrasound bath for 15 min. For solid samples containing 0.1% < TRIA < 1%, 100 mg of material were weighted and extracted with 500 μL of dimethyl sulfoxide and 25 mL of dichloromethane. After the adding of the IS, the solution was sonicated for 10 min. Extraction was performed twice, with further 20 mL of dichloromethane. If an aqueous layer was present, this was discharged using a separation funnel, and the organic layers were collected together. Finally, the organic phase was dried under vacuum at 45 °C and the solution was then dissolved in 5 mL of dichloromethane. For dried or fresh samples containing more than 1% of TRIA, 50 mg of material were weighed and, after the adding of IS, they were extracted with 500 μL of dimethyl sulfoxide and 25 mL of dichloromethane, as previously described. Finally, the organic layers were collected, dried under vacuum at 45 °C and the residue dissolved in 5 mL of dichloromethane.

#### 4.3.2. Aqueous Liquid Products

For aqueous samples containing less than 0.1% of TRIA, 50 mL of liquid were put in a separation funnel, added of the IS solution (100 μL of 500 µg/mL solution or absolute amounts of 300 to 500 μg of IS) and then extracted in a flask with dichloromethane (20 mL) for three times. The organic layer was collected, dried with sodium sulfate and finally evaporated under vacuum to 2 mL. For liquid samples with content of triacontanol > 0.1%, 10 mL of liquid were used, following the same protocol.

### 4.4. Chromatographic Conditions

An Agilent 1100 HPLC system (Agilent Technologies, Santa Clara, CA, USA) coupled to a Sedere Sedex 60 ELSD detector (Olivet, France) was used. In order to elute highly lipophilic compounds from the reverse phase column used as stationary phase (Agilent Extend C-18 4.6 × 150 mm, 5 µm), a gradient of acetonitrile (A) and methanol/methyl tertbutyl ether 10/90 (B) was used as mobile phase. Gradient conditions were optimized in order to perform the analysis in 30 min and to reach the best separation of TRIA and the IS. The gradient is reported in Table 5. Flow was 1 mL/min, injection volume was 10 µL.

**Figure 2:**
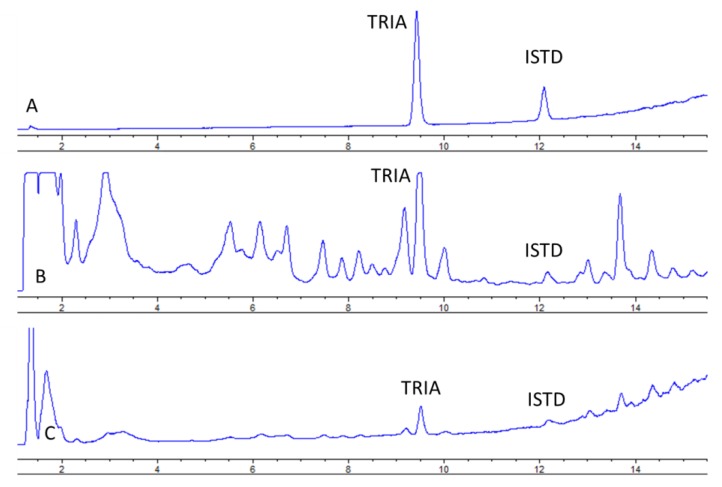
Chromatograms obtained from the analysis of (**A**) IST 5AC and TRIA 0.19% (*w*/*w*), (**B**) sample containing 200 mg/kg of TRIA and IST, (**C**) spiked sample containing 200 mg/kg TRIA and IST.

Under the proposed conditions, TRIA was eluted at 8.9 min (Figure 2) and peaks of both TRIA and IS were well resolved.

### 4.5. ELSD Conditions

Signal intensity in ELSD is influenced by pressure and temperature of nebulizer gas. Decreasing the temperature of the nebulizer from 70 °C to 40 °C yielded the improvement of signal-to-noise (S/N) ratio. Due to the low boiling point of the elution solvents used, a temperature of 40 °C appeared to be the best condition. Furthermore, the decrement of the nitrogen pressure from 2.2 to 1.1 bar also reduced the baseline noise, allowing the increase of the Limit of Detection (LOD) of all the analytes. At these conditions, TRIA and ISTD could be revealed up to 2 µg/mL.

### 4.6. Method Validation

The optimized method was validated according to the guidelines defined by the US Food and Drug Administration (FDA) [15]. Assay specificity was evaluated comparing the chromatograms of standard-spiked samples with standard solutions. Calibration curves were fitted by least square regression analysis to plot peak area ratio of TRIA/ISTD relatively to the ratio of the amount of TRIA/ISTD. Limit of Quantification (LOQ) was calculated as the lowest amount with a relative standard deviation < 20%. Intra and inter day stability, extraction recovery and matrix effects were measured. Precision and accuracy were evaluated using samples (n = 5) containing 10 to 5000 mg/kg of TRIA.

Calibration curves were prepared analyzing samples containing 0.10 < amount TRIA/IS < 10 in dichloromethane and plotting TRIA/IS AUC ratio versus TRIA/IS amount ratio. The ELSD response is not linear but follows a quadratic relationship, hence a quadratic calibration curve was obtained. The limit of detection (LOD) was established analyzing samples with known concentration of TRIA/IS and estimating the minimum concentration at which TRIA could be reliably detected (S/N ratio > 3, with RSD < 20%). On the other hand, LOQ for TRIA was estimated as the lowest concentration that gave an average S/N ratio > 10 (RSD < 20%). Intra-day and inter-day precisions were evaluated by analyzing TRIA/IS samples at TRIA concentration levels of 0.14–0.5% and 10 µg/kg five times within the same day as well as on two consecutive days, respectively.

## Figures and Tables

**Table 1:** Values used for the TRIA calibration curve.

Amount TRIA/IS	AUC TRIA/IS
0.10	0.02
0.50	0.15
1.00	0.43
2.00	1.52
5.00	5.08
7.00	10.13
10.00	16.84

**Table 2:** Intra-day and inter-day precision and accuracy at different concentrations.

Precision and Accuracy	Nominal Concentration (mg/kg)	Measured Concentration (mg/kg ± SD)	RSD (%)	Accuracy (%)
Intra-day (n = 5)	10	10.5 ± 0.7	11.2	105.0
	1400	1410 ± 2	1.70	100.5
	2600	2620 ± 4	8.33	100.9
	5000	5010 ± 7	1.44	100.1
Inter-day (n = 5)	10	10.1 ± 0.5	10.2	101.0
	1400	1410 ± 4	3.10	101.1
	2600	2610 ± 5	8.73	100.9
	5000	4990 ± 5	1.10	99.8

**Table 3:** Recovery of TRIA added to solid and liquid samples. TRIA was added to dried *Echinacea* roots and two samples of dried *M. sativa* to evaluate method recovery.

Sample	Final TRIA Amount in the Sample	Measured TRIA ± SD (n = 5)	% Recovery
*Echinacea* roots + TRIA	120 µg/g	119.8 ± 1.2 µg/g	99.8
*Echinacea* roots + TRIA	240 µg/g	237.8 ± 4.2 µg/g	99.1
*Echinacea* roots + TRIA	540 µg/g	525.8 ± 6.2 µg/g	97.2
*M. sativa*	270 µg/g	268.6 ± 3.1 µg/g	99.5
*M. sativa*	540 µg/g	530.1 ± 6.2 µg/g	98.3
*M. sativa* + TRIA	1300 µg/g	1300 ± 30 µg/g	100
*M. sativa* + TRIA	1900 µg/g	1880 ± 35 µg/g	99.0
TRIA solution	5 µg/mL	4.88 ± 0.10 µg/mL	97.6
TRIA solution	10 µg/mL	10.11 ± 0.12 µg/mL	101.0
TRIA solution	100 µg/mL	99.88 ± 0.32 µg/mL	99.9

**Table 4:** Comparison of the amounts of TRIA revealed by GC-MS and by HPLC-ELSD.

Sample	Values Measured by GC-MS (mg/kg)	Values Measured by HPLC-ELSD (mg/kg)
Dried *M. sativa* leaves	250 ± 11	262 ± 20
Dried *M. sativa* leaves and stems	130 ± 11	138 ± 11
Supercritical CO_2_ extract	4950 ± 50	5012 ± 80
Supercritical CO_2_ extract	28,050 ± 220	27,400 ± 300
Enzymatic extract of *M. sativa*	10 ± 1	10.10 ± 0.91
Enzymatic extract of *M. sativa* + supercritical CO_2_	15 ± 2	15.17 ± 1.31
Enzymatic extract of *M. sativa* + supercritical CO_2_	40 ± 2	42.90 ± 1.52

**Table 5:** HPLC gradient used for the analysis of TRIA.

Time (min)	% ACN	% MeOH-MTBE (10:90)
0–1	85	15
1–15	60	40
25–26	60	40
26–30	85	15
30	85	15

## Abbreviations

HPLC	High Performance Liquid Chromatography
ELSD	Evaporative Light Scattering Detector
TRIA	1-Triacontanol
5AC	5-α-Cholestane
EIC	Eicosanol
IS	Internal Standard
LOD	Limit of Detection
LOQ	Limit of Quantification

## References

[1] Naeem M., Khan M.M.A., Moinuddin (2012). Triacontanol: A potent plant growth regulator in agriculture. J. Plant Interact..

[2] Ries S. (1991). Triacontanol and its second messenger 9-β-l(+)-adenosine as plant growth substances. Plant Physiol..

[3] Ries S.K., Wert V. (1977). Growth responses of rice seedlings to triacontanol in light and dark. Planta.

[4] Li X., Zhong Q., Li Y., Li G., Ding Y., Wang S., Liu Z., Tang S., Ding C., Chen L. (2016). Triacontanol reduces transplanting shock in machine-transplanted rice by improving the growth and antioxidant systems. Front. Plant Sci..

[5] Jaybhay S., Chate P., Ade A. (2010). Isolation and identification of crude triacontanol from rice bran wax. J. Exp. Sci..

[6] Hwang K.T., Weller C.L., Cuppett S.L., Hanna M.A. (2004). Policosanol contents and composition of grain sorghum kernels and dried distillers grains. Cereal Chem..

[7] Dayan F.E., Cantrell C.L., Duke S.O. (2009). Natural products in crop protection. Bioorg. Med. Chem..

[8] Aftab T., Khan M.M.A., Idrees M., Naeem M., Singh M., Ram M. (2010). Stimulation of crop productivity, photosynthesis and artemisinin production in *Artemisia annua* L. by triacontanol and gibberellic acid application. J. Plant Interact..

[9] Ertani A., Schiavon M., Muscolo A., Nardi S. (2013). Alfalfa plant-derived biostimulant stimulate short-term growth of salt stressed *Zea mays* L. plants. Plant Soil.

[10] Yakhin O.I., Lubyanov A.A., Yakhin I.A., Brown P.H. (2016). Biostimulants in plant science: A global perspective. Front. Plant Sci..

[11] Harrabi S., Ferchichi A., Bacheli A., Fellah H. (2018). Policosanol composition, antioxidant and anti-arthritic activities of milk thistle (*Silybium marianum* L.) oil at different seed maturity stages. Lipids Health Dis..

[12] Wang C., Fan A., Zhu X., Lu Y., Deng S., Gao W., Zhang W., Liu Q., Chen X. (2015). Trace quantification of 1-triacontanol in beagle plasma by GC-MS/MS and its application to a pharmacokinetic study. Biomed. Chromatogr..

[13] Sierra R., González V.L., Magraner J. (2002). Validation of a gas chromatographic method for determination of fatty alcohols in 10 mg film-coated tablets of policosanol. J. AOAC Int..

[14] Kanya T.C.S., Rao L.J., Sastry M.C.S. (2007). Characterization of wax esters, free fatty alcohols and free fatty acids of crude wax from sunflower seed oil refineries. Food Chem..

[15] Food and Drug Administration, Center for Drug Evaluation and Research (CDER), Center for Veterinary Medicine (CVM), U.S. Department of Health and Human Services (2018). Bioanalytical Method Validation. Guidance for Industry. https://www.fda.gov/downloads/drugs/guidances/ucm070107.Pdf..

